# Patient-Reported Outcome Dashboards Within the Electronic Health Record to Support Shared Decision-making: Protocol for Co-design and Clinical Evaluation With Patients With Advanced Cancer and Chronic Kidney Disease

**DOI:** 10.2196/38461

**Published:** 2022-09-21

**Authors:** Laura M Perry, Victoria Morken, John D Peipert, Betina Yanez, Sofia F Garcia, Cynthia Barnard, Lisa R Hirschhorn, Jeffrey A Linder, Neil Jordan, Ronald T Ackermann, Alexandra Harris, Sheetal Kircher, Nisha Mohindra, Vikram Aggarwal, Rebecca Frazier, Ava Coughlin, Katy Bedjeti, Melissa Weitzel, Eugene C Nelson, Glyn Elwyn, Aricca D Van Citters, Mary O'Connor, David Cella

**Affiliations:** 1 Department of Medical Social Sciences Northwestern University Feinberg School of Medicine Chicago, IL United States; 2 Robert H Lurie Comprehensive Cancer Center Northwestern University Feinberg School of Medicine Chicago, IL United States; 3 Department of Psychiatry & Behavioral Sciences Northwestern University Feinberg School of Medicine Chicago, IL United States; 4 Northwestern Medicine Northwestern University Feinberg School of Medicine Chicago, IL United States; 5 Division of General Internal Medicine Department of Medicine Northwestern University Feinberg School of Medicine Chicago, IL United States; 6 Robert J Havey, MD Institute for Global Health Northwestern University Feinberg School of Medicine Chicago, IL United States; 7 Institute for Public Health and Medicine Northwestern University Feinberg School of Medicine Chicago, IL United States; 8 Center of Innovation for Complex Chronic Healthcare Hines VA Hospital Hines, IL United States; 9 Division of Hematology and Oncology Department of Medicine Northwestern University Feinberg School of Medicine Chicago, IL United States; 10 Division of Nephrology Department of Medicine Northwestern University Feinberg School of Medicine Chicago, IL United States; 11 The Dartmouth Institute for Health Policy & Clinical Practice Geisel School of Medicine Dartmouth College Hanover, NH United States

**Keywords:** patient-reported outcome measures, shared decision-making, medical informatics, coproduction, learning health system, cancer, chronic kidney disease

## Abstract

**Background:**

Patient-reported outcomes—symptoms, treatment side effects, and health-related quality of life—are important to consider in chronic illness care. The increasing availability of health IT to collect patient-reported outcomes and integrate results within the electronic health record provides an unprecedented opportunity to support patients’ symptom monitoring, shared decision-making, and effective use of the health care system.

**Objective:**

The objectives of this study are to co-design a dashboard that displays patient-reported outcomes along with other clinical data (eg, laboratory tests, medications, and appointments) within an electronic health record and conduct a longitudinal demonstration trial to evaluate whether the dashboard is associated with improved shared decision-making and disease management outcomes.

**Methods:**

Co-design teams comprising study investigators, patients with advanced cancer or chronic kidney disease, their care partners, and their clinicians will collaborate to develop the dashboard. Investigators will work with clinic staff to implement the co-designed dashboard for clinical testing during a demonstration trial. The primary outcome of the demonstration trial is whether the quality of shared decision-making increases from baseline to the 3-month follow-up. Secondary outcomes include longitudinal changes in satisfaction with care, self-efficacy in managing treatments and symptoms, health-related quality of life, and use of costly and potentially avoidable health care services. Implementation outcomes (ie, fidelity, appropriateness, acceptability, feasibility, reach, adoption, and sustainability) during the co-design process and demonstration trial will also be collected and summarized.

**Results:**

The dashboard co-design process was completed in May 2020, and data collection for the demonstration trial is anticipated to be completed by the end of July 2022. The results will be disseminated in at least one manuscript per study objective.

**Conclusions:**

This protocol combines stakeholder engagement, health care coproduction frameworks, and health IT to develop a clinically feasible model of person-centered care delivery. The results will inform our current understanding of how best to integrate patient-reported outcome measures into clinical workflows to improve outcomes and reduce the burden of chronic disease on patients and health care systems.

**International Registered Report Identifier (IRRID):**

DERR1-10.2196/38461

## Introduction

### Background

**Figure 1:**
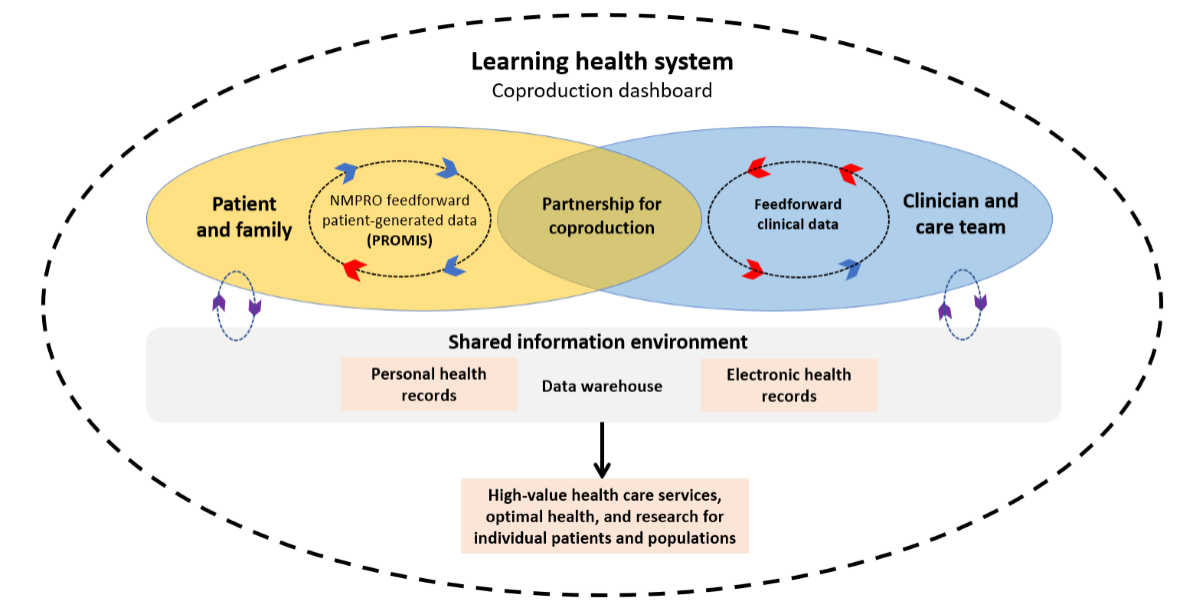
Dartmouth coproduction model for clinical integration of patient-reported outcome measures (adapted from Nelson et al [17]). NMPRO: Northwestern Medicine Patient-Reported Outcomes; PROMIS: Patient-Reported Outcomes Measurement Information System.

Over half of Americans are currently living with a chronic illness such as cancer or chronic kidney disease [1,2], and supporting their symptom and care management needs is an urgent priority. Most patients living with a chronic illness experience distressing symptoms and side effects, most commonly fatigue, pain, and emotional distress such as fear or sadness [3-5]. Furthermore, these symptoms often go unrecognized by clinicians during regular visits [6,7], leading patients to experience chronically unmanaged symptoms that can escalate and increase the risk of potentially avoidable health care use [8,9]. Following distress screening guidelines from multiple organizations such as the Centers for Medicare and Medicaid Services [10,11], the National Comprehensive Cancer Network [12], and the Commission on Cancer [13], health care systems have begun to implement patient-reported outcome measures to facilitate more timely identification of symptom management needs. The most common strategies for implementing patient-reported outcome measures in clinical care involve feedback of results to clinicians to trigger clinical action such as referrals or feedback to patients to promote self-management of symptoms [14,15]. In this study, we will co-design and pilot-test an alternative system that will feed patient-reported outcome data back to patients *and* clinicians simultaneously through a shared interface to be used primarily during health care encounters (Figure 1). If implemented successfully, our approach has the potential to improve patient well-being and other clinical outcomes by supporting a collaborative health care communication and shared decision-making process [16-18].

Efforts to improve care delivery for advanced cancer and chronic kidney disease offer strong opportunities to develop and evaluate strategies for implementing patient-reported outcome measures. Medical advances in treatments have made it possible to live months or years with these diagnoses, meaning that patients need to manage their symptoms and treatments on a regular basis and across ongoing health care encounters [19,20]. Both diagnoses are accompanied by significant symptom burden [3], putting patients at risk of decreased health-related quality of life and increased health care expenditures. Currently, national costs associated with cancer and chronic kidney disease exceed US $100 billion per year, with most of those expenditures occurring during the advanced stages of both illnesses [21-23]. In addition, patients need to navigate complex and preference-sensitive decisions about their health care that involve considering their health-related quality of life priorities [24,25]. For instance, the decision to select treatments focused on extending life (eg, chemotherapy and dialysis) versus treatments focused on optimizing comfort (eg, best supportive care) requires thoughtful consideration of how one will feel in the future, both physically and emotionally, in response to treatment options [10,26]. Regularly monitoring patient-reported outcomes can also decrease the use of low-value health care services by creating opportunities for patients to discuss bothersome side effects with their clinicians and revisit alternative treatment options that might provide more value [10,27]. Therefore, incorporating patient-reported outcome measures into care for advanced cancer and chronic kidney disease can improve patients’ health-related quality of life, align care provision with patient preferences and needs, and decrease the burden on the health care system by reducing unnecessary costly services.

Given these benefits, patient-reported outcome data can enhance the health care *coproduction* process [16]. Coproduction is an approach to health care delivery that posits that health care services and outcomes result from an interaction between clinician- and consumer-driven factors; thus, health care should be *coproduced* by patients, families, and health care professionals to achieve optimal health [16,28]. At the individual level, health care coproduction includes empowering patients and clinicians to *coassess* health status, *coplan* health care decisions, *co-design* acceptable and effective health services, and ultimately *co-deliver* those services [16,28]. As health IT has advanced, proponents have suggested that health care systems can encourage coproduction by implementing data visualization dashboards that are linked to the electronic health record and are accessible to both patients and clinicians [17,18,29,30]. These dashboards can display patient-generated data on symptoms, health-related quality of life, and goals of care along with key clinical data, allowing patients, care partners, and clinicians to view and discuss information together during and after scheduled visits (Figure 1). During in-clinic evaluations, patient-reported outcome clinical dashboards have demonstrated feasibility and acceptability, and preliminary evidence shows that dashboard users report higher-quality shared decision-making, a key process of coproduction, compared with nonusers [15,30-32]. However, no previous study has evaluated whether these dashboards are associated with changes from baseline to follow-up time points in shared decision-making and related disease management outcomes.

### Objectives

This paper details the protocol for our study. We will design, implement, and longitudinally evaluate a clinician- and patient-facing dashboard for displaying patient-reported outcomes and other data from the electronic health record. The dashboard will be co-designed by patients and care partners, clinicians, and researchers to optimize the likelihood that it will be acceptable, feasible, and effective [33]. By fully integrating relevant information in a way that is easily viewed, discussed, and revisited by patients and clinicians, the dashboard may also promote other coproduction components such as symptom coassessment, coplanning through shared decision-making, and codelivery of health services by activating and empowering patients to self-manage their symptoms and care [16]. Although the study’s primary focus is on enhancing the coproduction process for individual patients, the study falls within a quality improvement initiative that ultimately aims to achieve coproduction at the health care system level as well; situated within a learning health system, the dashboard implementation will continuously collect aggregated patient- and clinician-generated data to ensure that stakeholder perspectives are represented in system-wide policy decisions [17,29,34]. Therefore, the dashboard has the potential to improve health care system–level outcomes and care efficiency.

This study has two objectives: (1) to co-design a clinical dashboard that integrates patient-reported outcomes with other clinical data (eg, vital signs, laboratory tests, and medications) through the electronic health record and (2) to conduct a longitudinal demonstration trial evaluating whether the dashboard is associated with improved shared decision-making and disease management outcomes. Within objective 2, we hypothesize that patients who use the dashboard will experience increases from baseline to the 3- and 6-month follow-ups in perceived quality of shared decision-making, satisfaction with care, self-efficacy in managing treatments and symptoms, and health-related quality of life. We also hypothesize that, compared with historically and demographically matched controls, patients who use the dashboard will have a greater reduction in use of potentially avoidable, high-cost, and low-value health care services (see the Methods section for specific indicators that will be tested). By carefully delineating the dashboard design and evaluation process, this study will enable the dashboard’s scalability and inform future investigations aiming to adapt the patient-reported outcome dashboard to other health care systems.

## Methods

### Ethics Approval

The Northwestern University Institutional Review Board approved all procedures described in this manuscript and a detailed data security plan (STU00210091, STU00211654, and STU00212634). Depending on the assessment point, survey data will be collected and stored securely through the health care system’s electronic patient portal or through a REDCap (Research Electronic Data Capture; Vanderbilt University) [35] server hosted at the Northwestern University Feinberg School of Medicine, both of which are protected by firewalls. Health service use data will be extracted from the Northwestern Medicine Enterprise Data Warehouse by trained Northwestern Medicine data analysts and entered into the study’s REDCap database by the study coordinator (AC) approved by the ethics committee. Audio-recorded focus group data will be collected and stored in compliance with the Health Insurance Portability and Accountability Act. Audio files will be transcribed and deidentified, and transcriptions will be stored on a secure and password-protected Northwestern University Feinberg School of Medicine server accessible only to research staff listed on the study protocol approved by the ethics committee. After the transcription process is complete, all audio files will be deleted. All study procedures were considered low-risk by the Northwestern University Institutional Review Board, and the ethics review concluded that the benefits outweighed any minimal risks.

### Objective 1: Dashboard Co-design Process

#### Clinical Setting

The dashboard will build on an existing infrastructure called the Northwestern Medicine Patient-Reported Outcomes system. This is the health care system’s current technological framework for administering patient-reported outcome measures electronically and integrating data into the electronic health record to inform clinical care delivery [36]. Specifically, patients receive an email alert through their electronic patient portal 72 hours before an upcoming appointment, prompting a response to a patient-reported outcome questionnaire about symptoms and supportive care needs. To optimize measurement precision using as few items as possible, measures from the Patient-Reported Outcomes Measurement Information System (PROMIS) are used to assess anxiety, depression, pain, fatigue, and physical function. The electronic questionnaire automatically scores the patients’ responses, stores them in the electronic health record, and generates alerts to their care team for any endorsed needs or clinically elevated symptoms.

In this study, this system will be adapted, with patient and other stakeholder input, into a clinical dashboard to facilitate symptom management and shared decision-making during scheduled health care visits for patients with advanced cancer and chronic kidney disease. Patient and care partner input will maximize the dashboard’s patient-centeredness. By including clinicians in the design process, attention will be paid to the possible impact of dashboard participation on digital fatigue. To direct attention to and encourage discussion of the more potentially bothersome “hard-stop” alerts, alert thresholds on the patient-reported outcome measures will be set relatively high. The dashboard will be viewable by both patients (via the electronic patient portal) and physicians (via the health system’s electronic health record software) and will automatically populate electronic health record–linked data, including patient-reported outcomes, laboratory test results, medications, and vital signs.

#### Participants and Eligibility Criteria

The first objective of the study is to collaborate with stakeholders who represent “end users” to co-design our clinical dashboard. End users include patients with advanced cancer or advanced chronic kidney disease, their care partners, and their clinicians. Clinician participants will include 2 oncologists (specializing in the treatment of gastrointestinal and lung cancer), a nephrologist, a nephrology physician assistant, and 2 primary care physicians. Patients will be eligible to participate if they have a history of receiving care from one or more of the participating clinicians at Northwestern Memorial Health Care. Patients with gastrointestinal cancer must have a confirmed diagnosis of stage 4 gastrointestinal cancer and have been receiving intravenous chemotherapy for at least three months. Patients with lung cancer must have a confirmed diagnosis of stage 3C or 4 lung cancer and have been receiving first- or second-line chemotherapy for at least 3 months. Patients with chronic kidney disease must have a confirmed diagnosis of at least stage 3 (defined as a clinical diagnosis or an estimated glomerular filtration rate of <60). Care partners will be eligible to participate if they assist in the care of a patient who would meet the inclusion criteria.

#### Procedures

**Figure 2:**
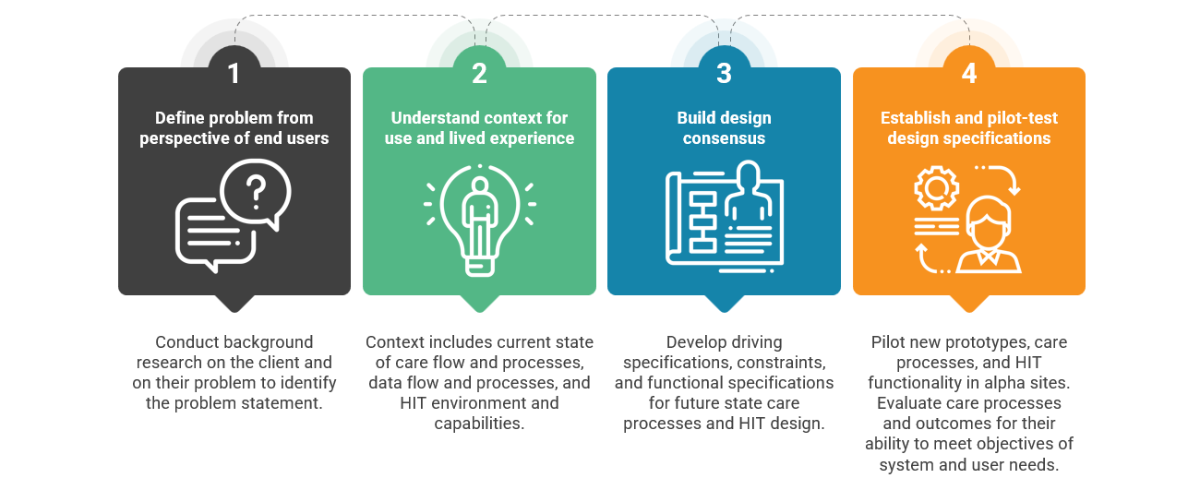
Dartmouth model for dashboard co-design and implementation (reproduced from Coproduction Design and Implementation Flow by Van Citters [37], which is published under Creative Commons Attribution 4.0 International License [39]). HIT: health IT.

For each of the two disease groups of interest (advanced cancer and chronic kidney disease), we will convene a co-design team comprising approximately 20 highly engaged stakeholders, including investigators, patients, care partners, clinicians, and health IT professionals. Co-design teams will iteratively develop the dashboard over the course of monthly meetings held during the first year of the study. Co-design meetings will involve team-based working sessions with predefined objectives and deliverables for each meeting (Table 1). The co-design process will occur in 4 broad phases informed by the Dartmouth Model for Co-design and Implementation (Figure 2 [37,38]), which has been successfully used to co-design and implement coproduction dashboards for people living with other chronic illnesses [31,32]. The 4 phases of co-design will include (1) defining the problem from the perspective of the end users, (2) understanding the context of use and lived experience, (3) building a design consensus, and (4) establishing and pilot-testing design specifications [37].

**Table 1:** Detailed outline of dashboard co-design session objectives.

Activity	Co-design phase (Figure 2)	Objectives
Co-design launch meeting	1	Introduction to the project and key concepts Review of co-design working structure and scope of work
Working session 1	1 and 2	Team building Identifying facilitators of and barriers to shared decision-making and health coproduction Exploring how better sharing of information can help
Working session 2	2	Developing an understanding of how information can address facilitators of and barriers to improving care management
Working session 3	2 and 3	Identifying common themes, priorities, and values regarding information and shared decision-making of dashboard end users Developing team-specific co-design objectives
Working session 4	1, 2, and 3	Refining team-specific objectives Introducing options for data elements to populate dashboards Envisioning dashboard
Working session 5	2, 3, and 4	Exploring the use of a dashboard in a case example to advance emerging dashboard concepts
Working session 6	2, 3, and 4	Exploring the use of a dashboard in a case example focused on a point of shared decision-making to advance emerging dashboard concepts
Working session 7	2 and 3	Defining priority dashboard elements by dashboard user type Proposing questions for external validation (focus groups) Exchanging ideas and plans for dashboard concept between cancer and kidney disease teams
Working session 8	2 and 3	Reviewing dashboard drafts and confirming alignment with co-design teams’ visions Reviewing data sources and measures to populate dashboards
Working session 9	2 and 3	Reviewing feasible dashboard display options Confirming completeness and appropriateness of planned data elements
Working session 10	2 and 3	Demonstration of programmed dashboard display Cancer and kidney disease co-design teams present respective dashboards and exchange ideas.
Working session 11	3 and 4	Demonstration and critical review of fully programmed dashboards
Working session 12	2 and 3	Interactive demonstration of fully programmed dashboards and questionnaires Establishing specifications for alerts (symptom thresholds and routing) Determining communication strategy and framing of patient-facing questionnaires
Working session 13 (physician champion working meeting)	2, 3, and 4	Confirming final dashboard specifications Demonstration and discussion of in-basket alerts Confirming final patient dashboard user criteria Confirming implementation workflows
Co-design wrap-up meeting	4	Live demonstration of the prefinal dashboards and questionnaires Conducting a reflection on the entire design process with respect to participation in co-design activities Examining and discussing implications of COVID-19 and considerations for telehealth

Table 1 provides a detailed outline of the objectives of each co-design meeting. In phase 1 of the co-design process (defining the problem from the perspective of the end users), the investigators will introduce team members to the project and to the concepts of health care coproduction and shared decision-making. Team members will brainstorm facilitators of and barriers to shared decision-making and optimal care planning. In phase 2 (understanding the context of use and lived experience), co-design teams will discuss current clinical workflows for patient-reported outcome assessment and shared decision-making, brainstorm challenges and opportunities for solutions, and document end users’ priorities for the dashboard’s design and features. In phase 3 (building a design consensus), co-design meetings will focus on drafting, discussing, and revising functional mock-ups of the dashboard, including design specifications and which clinical and patient-reported outcomes should be incorporated. Although patients will inform the specific symptoms and patient-reported outcomes that are important to assess, the investigators will select the appropriate measures for each outcome based on their measurement expertise. These will include measures from the Functional Assessment of Chronic Illness Therapy [39], PROMIS [40], and the Patient-Reported Outcomes version of the Common Terminology Criteria for Adverse Events (PRO-CTCAE) [41]. In phase 4 (establishing and pilot-testing design specifications), co-design teams will provide iterative user experience feedback on the dashboard until its usability, feasibility, and compatibility with the electronic health record and clinical workflow systems are optimized. After each co-design meeting, the participants will be sent a brief survey via email to provide additional feedback.

In parallel to these intensive co-design team meetings, we will also hold supplemental focus groups. The primary purpose of these focus groups is to validate the evolving consensus and work product of the co-design teams with additional stakeholders. In tandem with phases 1 and 2 of co-design, we will conduct a focus group with 24 patients and care partners to gather an initial assessment of attitudes, beliefs, and perceptions to inform the dashboard’s development. In tandem with phase 3 of co-design, we will conduct focus groups with a total of 72 stakeholders separated by disease group (cancer vs chronic kidney disease) and role (patients and care partners vs clinicians) to provide additional feedback on the appropriateness and desirability of the proposed dashboard design and content. In tandem with phase 4 of co-design, we will conduct focus groups to confirm the acceptability and usability of the dashboard (n=16-24 patients and care partners per disease group). Trained members of the research team will facilitate the discussions according to guides containing semistructured questions to follow in each session. Research staff will take notes during these sessions, which will also be audio recorded in case project staff are unable to manually document all responses.

#### Outcomes

The primary outcome of the co-design process is to build a fully functional dashboard that can be implemented for clinical testing in objective 2 of this study. However, we also anticipate the development of a rich source of quantitative and qualitative data on the dynamics of development and implementation outcomes [42]. Data sources will include recordings and notes from the co-design meetings, focus groups, and postmeeting surveys. *Appropriateness* will capture stakeholders’ perceptions of the compatibility between the proposed dashboard and the target clinical setting’s goals and needs, whereas *feasibility* will assess perceptions of whether and how it can be successfully implemented into the clinical infrastructure. These 2 outcomes will be captured qualitatively through phase 1 and 2 co-design meetings and focus groups as well as through quantitative and qualitative responses provided by clinicians in a prelaunch survey conducted just before dashboard implementation. The *acceptability* of the near-final dashboard will reflect stakeholders’ satisfaction with its design, content, and functionality. This outcome will be captured qualitatively through feedback provided during co-design meetings, co-design postmeeting surveys, and phase 4 focus groups’ usability evaluations of the co-designed dashboard. The *fidelity* of the co-design process, or how well it was carried out as intended, will be assessed quantitatively through responses to postmeeting evaluations sent to each member of the co-design teams.

#### Analyses

Each of the outcomes will be summarized through qualitative analysis or descriptive statistics depending on the data type. For qualitative analyses, transcripts and notes from the sessions will be analyzed using a directed content analysis approach [43] guided by two established implementation science frameworks: the Consolidated Framework for Implementation Research [44] and the Reach, Effectiveness, Adoption, Implementation, and Maintenance framework [45]. We will use a combination of traditional qualitative analysis and rapid qualitative analysis [46,47], which will allow investigators to provide rapid feedback to operational partners to inform the dashboard building and implementation processes. Across the study phases, qualitative analyses will be conducted by approximately 5 to 7 coders trained in qualitative methods and relevant implementation frameworks. The team will establish coding frameworks or dictionaries (eg, by all coding an initial transcript, discussing and refining codes, and reaching a consensus about themes and categories). The remaining transcripts will then be double-coded and supervised by a lead team member. The full team will meet to discuss the final results to resolve any questions and elucidate the resultant themes. These strategies will be used to aggregate and summarize the main points and evaluate the relevant implementation outcomes (appropriateness, feasibility, and acceptability).

### Objective 2: Demonstration Trial

#### Overview

**Figure 3:**
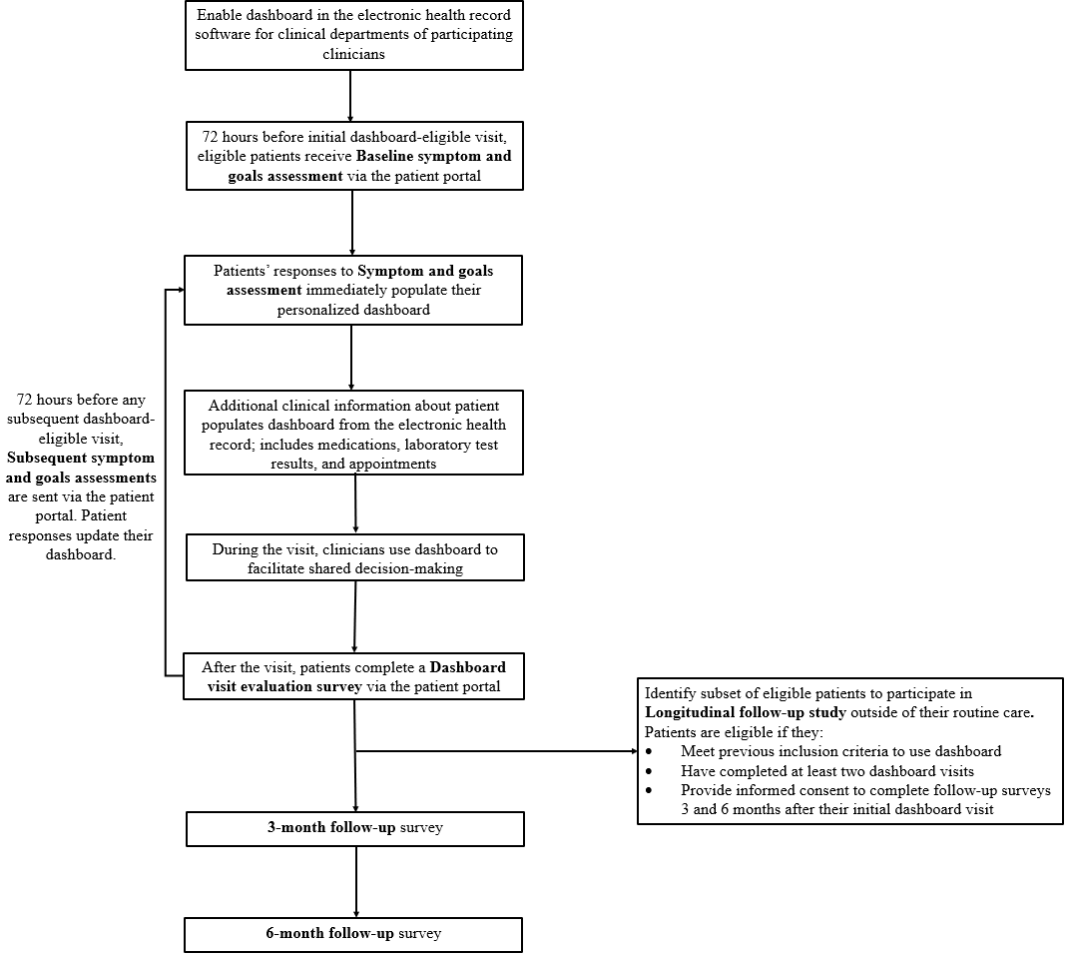
Demonstration trial flow diagram.

The second objective of this study will include testing the co-designed dashboard in the flow of clinical care service delivery. First, as a quality improvement initiative, the dashboard will be implemented at the clinic level with all eligible patients who receive care from participating clinicians. This step will allow us to collect clinic-wide quality metrics associated with dashboard implementation. Second, the project will include a longitudinal follow-up study among a subset of patients to assess changes in patient outcomes associated with using the dashboard. Figure 3 shows the flow diagram of the dashboard’s clinical implementation and evaluation. Table 2 provides a schedule of the survey measures completed by the patients at each assessment point of the demonstration trial. These procedures and measures are described in more detail in the following sections.

**Table 2:** Schedule of survey measures in demonstration trial^a^.

Domain and subdomains				Baseline symptom and goals assessment		Subsequent symptom and goals assessments		Dashboard visit evaluation		3-month follow-up		6-month follow-up
**Patient measures**												
	Sociodemographic data			Age, gender, race, ethnicity, marital status, employment, and education		—^b^		—		—		—
	Goals of care			De novo measure		De novo measure		—		—		—
	**Health-related quality of life**											
		Health perception	PROMIS^c^ Global01 [40]		PROMIS Global01		—		—		—	
		Global	FACT-G7^d^ [48]		FACT-G^e^ GF7^f^ item [48]		—		FACT-G7		FACT-G7	
		Fatigue	PROMIS 2-item custom SF^g^ [49]		PROMIS 2-item custom SF		—		PROMIS CAT^h^ [49]		PROMIS CAT	
		Anxiety	PROMIS 2-item custom SF [50]		PROMIS 2-item custom SF		—		PROMIS CAT [50]		PROMIS CAT	
		Pain	PROMIS 2-item custom SF [51]		PROMIS 2-item custom SF		—		PROMIS CAT [51]		PROMIS CAT	
		Depression	PROMIS 2-item custom SF [50]		PROMIS 2-item custom SF		—		PROMIS CAT [50]		PROMIS CAT	
		Physical function	PROMIS 2-item custom SF [52]		PROMIS 2-item custom SF		—		PROMIS CAT [52]		PROMIS CAT	
		Shortness of breath	PROMIS DYSSV014 item [53]		PROMIS DYSSV014 item		—		—		—	
		Urinary frequency (chronic kidney disease only)	PRO-CTCAE^i^ 63^a^ item [41]		PRO-CTCAE 63^a^ item		—		—		—	
		Edema	PRO-CTCAE 22^b^ item [41]		PRO-CTCAE 22^b^ item		—		—		—	
		Nausea	PROMIS GISX49 item [54]		PROMIS GISX49 item		—		—		—	
		Appetite	PROMIS GISX55 item [54]		PROMIS GISX55 item		—		—		—	
		Itching (chronic kidney disease only)	PROMIS PIQSeverity04 item [55]		PROMIS PIQSeverity04 item		—		—		—	
		Neuropathy (cancer only)	FACT^j^ and GOG-NTX-4^k^ (version 4) [56]		FACT and GOG-NTX-4 (version 4)		—		—		—	
		Constipation (cancer only)	PRO-CTCAE 15^a^ item [41]		PRO-CTCAE 15^a^ item		—		—		—	
		Diarrhea (cancer only)	PROMIS GISX38 item [54]		PROMIS GISX38 item		—		—		—	
		Side effect bother	FACT-G GP5^l^ item [39]		FACT-G GP5 item		—		—		—	
	Shared decision-making			CollaboRATE [57]		—		CollaboRATE		CollaboRATE		CollaboRATE
	Self-efficacy-managing treatments			PROMIS 4-item custom SF [58]		—		—		PROMIS 4-item custom SF		PROMIS 4-item custom SF
	Self-efficacy-managing symptoms			PROMIS 3-item custom SF [58]		—		—		PROMIS 3-item custom SF		PROMIS 3-item custom SF
	Treatment satisfaction			FACIT-TS^m^ TS40 item [59]		—		FACIT-TS TS40 item		FACIT-TS TS40 item		FACIT-TS TS40 item
	Treatment satisfaction (cancer only)			CAHPS^n^ Cancer Care Survey [60]		—		—		CAHPS Cancer Care Survey		CAHPS Cancer Care Survey
	Health care communication			CASE^o^ (information factor) [61]		—		—		CASE (information factor)		CASE (information factor)
	Medication adherence			PMAS^p^ [62]		—		—		PMAS		PMAS
	Social isolation			PROMIS UCLA^q^ 14x2 item [63]		—		—		PROMIS UCLA 14x2 item		PROMIS UCLA 14x2 item
	Health literacy			SILS^r^ [64]		—		—		SILS		SILS
	Financial toxicity			COST^s^-FACIT^t^ FT12 item [65]		—		—		COST-FACIT FT12 item		COST-FACIT FT12 item
	Usability, acceptability, and adoption			—		—		—		SUS^u^ [66], SPHERE^v^ [67], and NoMAD^w^ [68]		SUS, SPHERE, and NoMAD
	Fidelity of dashboard use			—		—		De novo item		—		—
**Clinician measures**												
	Usability, acceptability, and adoption			—		—		—		SUS, SPHERE, and NoMAD		SUS, SPHERE, and NoMAD
	Fidelity of dashboard use			—		—		—		De novo item		De novo item
	Dashboard sustainability			—		—		—		—		CSAT^x^ [69]

^a^Single-item names were obtained from the referenced parent measures or item banks.

^b^Empty cells indicate that a given domain was not included at that particular assessment point.

^c^PROMIS: Patient-Reported Outcomes Measurement Information System.

^d^FACT-G7: Functional Assessment of Cancer Therapy-General, 7-item version.

^e^FACT-G: Functional Assessment of Cancer Therapy-General.

^f^GF7: FACT-G global quality of life item.

^g^SF: short form.

^h^CAT: computerized adaptive test.

^i^PRO-CTCAE: Patient-Reported Outcomes version of the Common Terminology Criteria for Adverse Events.

^j^FACT: Functional Assessment of Cancer Therapy.

^k^GOG-NTX-4: Gynecologic Oncology Group-Neurotoxicity.

^l^FACT-G GP5: FACT-G side effect bother item.

^m^FACIT-TS: Functional Assessment of Chronic Illness Therapy-Treatment Satisfaction.

^n^CAHPS: Consumer Assessment of Health Plans Study.

^o^CASE: Communication and Attitudinal Self-Efficacy scale.

^p^PMAS: PROMIS Medication Adherence Scale.

^q^UCLA: University of California, Los Angeles.

^r^SILS: Single Item Literacy Screener.

^s^COST: Comprehensive Score for Financial Toxicity.

^t^FACIT: Functional Assessment of Chronic Illness Therapy.

^u^SUS: System Usability Scale.

^v^SPHERE: Stroke Prevention in Healthcare Delivery Environments study.

^w^NoMAD: Normalization Measure Development.

^x^CSAT: Clinical Sustainability Assessment Tool.

#### Procedures

##### Clinic-Wide Dashboard Implementation

The co-designed dashboard will be enabled in the health system’s electronic health record software within the clinical departments of all participating clinicians. The first month of dashboard implementation will constitute a “soft launch” period where participating clinicians and their eligible patients will be trained on using the dashboard and will work with the project team to troubleshoot and resolve any technical and practical issues. After the soft launch period, clinicians will be encouraged to use the dashboard during clinical encounters with all their patients who meet the inclusion criteria (as described previously in objective 1). As outlined in Figure 3, eligible patients will receive a baseline symptom and goals assessment (see Table 2 for specific measures) through the electronic patient portal approximately 72 hours before their initial dashboard-eligible visit. If a patient does not respond to the symptom and goals assessment at home, the study staff will assist patients to complete it over the telephone or in-clinic before the appointment. Responses to the symptom and goals assessment, in addition to their most recent results on other clinical data, will populate the dashboard in real time. Clinicians will use the dashboard during the upcoming visit to improve communication and shared decision-making with patients. Following the visit, patients will receive a dashboard visit evaluation survey through the electronic patient portal to provide feedback on their care experience and the shared decision-making process that occurred during their visit. This process will be repeated for any subsequent visit that a patient has with their participating clinician during the study period.

##### Dashboard Content

The dashboard will display results from the patient’s most recent symptom and goals assessment and other clinical measures stored in the electronic health record. The included assessments will be determined through extensive stakeholder input during the dashboard co-design process described in objective 1. The symptom and goals assessment will include a mix of items and scales selected to optimize clinical relevance and feasibility. For instance, each assessment will include the PROMIS Global01 health perception item [40], the Functional Assessment of Cancer Therapy-General (FACT-G) GF7 global quality of life item [39], and the FACT-G GP5 side effect bother item [39]. Two-item PROMIS custom short forms will assess anxiety [50], depression [50], fatigue [49], pain [51], and physical function [52]. Additional symptoms will mostly be assessed with single items, for example, the PROMIS DYSSV014 dyspnea item [53], the PRO-CTCAE 63a urinary frequency item (chronic kidney disease only) [41], or the PRO-CTCAE 15a constipation item (cancer only) [41]. Table 2 provides a complete list of health-related quality of life measures to be included in each symptom and goals assessment, and the results will be displayed within the dashboard in a user-friendly format.

In addition to rating health-related quality of life domains, patients will respond to 5 open-response questions in the symptom and goals assessment regarding their goals of care that will populate the dashboard. These questions will prompt patients to specify (1) the top 1 or 2 concerns they would like to discuss during the visit, (2) their most concerning side effects, (3) overall goals regarding their cancer or kidney disease treatment, (4) personal goals and values, and (5) how they can work together with their care team to achieve their goals. Finally, the dashboard will display the most recent results on other clinical measures typically collected during the care process and stored within the electronic health record. For patients with cancer, the additional clinical data will include medications, weight, white blood cell count, absolute neutrophils, hemoglobin, albumin, and an appointment schedule. For patients with chronic kidney disease, the additional clinical data will include weight, blood pressure, glomerular filtration rate, hemoglobin A1c, microalbuminuria, urine protein, and an appointment schedule. Patients and clinicians will view the patient’s individualized dashboard on a computer screen during the visits. Patients will also be able to view their dashboard in between visits using their own devices (eg, computer, tablet, and mobile phone) that can access their electronic patient portal.

##### Longitudinal Follow-up Study

To assess the longitudinal changes in our primary and secondary study outcomes, a subset of approximately 200 patients will participate in a longitudinal follow-up study. Patients will be eligible to participate if they meet all other study inclusion criteria, have completed at least two dashboard-eligible visits, and provide informed consent to complete a follow-up survey at 3 and 6 months. In parallel to collecting these follow-up patient outcomes, all participating clinicians will also complete surveys at 3 and 6 months to report on their experience with the dashboard’s implementation (Table 2). All survey data collected during the longitudinal follow-up study will be collected outside the electronic patient portal using a web-based survey platform (REDCap) [35].

##### Dashboard-Naïve Comparison Group

For analyses focusing on health service use (see outcome description in the Health Service Use section), we will identify two cohorts of patients who were not exposed to the dashboard: (1) a cohort of eligible patients with cancer and chronic kidney disease who received care concurrently from participating Northwestern Medicine clinicians but who did not enroll in the dashboard study and (2) a matched cohort of patients with cancer and chronic kidney disease treated contemporaneously by nonparticipating Northwestern Medicine clinicians. Health service use variables will be the only study outcome data available for these 2 comparison groups.

#### Primary Study Outcome

The central outcome of the study is shared decision-making in the treatment of patients with advanced cancer or chronic kidney disease. We will use the CollaboRATE measure [57] to assess patients’ perceptions of the quality of shared decision-making occurring in patient-clinician interactions. Patients will complete the measure at baseline, 3-month follow-up, and 6-month follow-up. In addition, they will respond to the CollaboRATE measure in each dashboard visit evaluation survey to assess the perceived quality of shared decision-making that occurred during each dashboard visit. CollaboRATE is a validated 3-item measure that was developed with significant patient input and asks patients to rate the extent to which their clinicians helped them understand health issues, how much effort was made to listen to their priorities, and how much effort was made to include their priorities in treatment selection. We will use the 5-category response scale version (0=“No effort was made,” 1=“A little effort was made,” 2=“Some effort was made,” 3=“A lot of effort was made,” and 4=“Every effort was made”). Scores will be generated by summing responses to the 3 items and generating a score ranging from 0 to 12, with higher scores indicating greater shared decision-making. The CollaboRATE measure is generic and, therefore, it is appropriate for use with patients with various chronic conditions.

#### Secondary Study Outcomes

##### Satisfaction With Health Care Quality

Satisfaction with health care will be assessed at baseline, 3 months, and 6 months with a single item from the Functional Assessment of Chronic Illness Therapy-Treatment Satisfaction measure [59]. Patients will also respond to the item in each dashboard visit evaluation survey to assess their satisfaction with the dashboard visit. Patients will complete item TS40—“How do you rate the care you received?”—on a scale of 0 (poor) to 4 (excellent). The scale has met psychometric standards for a variety of chronic health conditions [59]. In addition, 5 items from the Consumer Assessment of Health Plans Study Cancer Care Survey, Drug Therapy Version [60], will be assessed at baseline, 3 months, and 6 months among patients with cancer only. These items measure patients’ experiences with cancer care. For example, the item “In the last 6 months, did your drug therapy team advise you about or help you deal with these changes in your energy levels?” is rated as 1 (Yes, definitely), 2 (Yes, somewhat), or 3 (No).

##### Self-efficacy for Managing Chronic Conditions

A total of 2 subdomains of the PROMIS Self-Efficacy for Managing Chronic Conditions domain will be assessed at baseline and at the 3- and 6-month follow-ups. A 4-item custom short form created from the PROMIS Self-Efficacy for Managing Medications and Treatment Item Bank version 1.0 [58] will assess patients’ confidence in their ability to follow treatment and medication plans. A 3-item custom short form created from the PROMIS Self-Efficacy for Managing Chronic Conditions-Managing Symptoms Item Bank version 1.0 [58] will assess confidence in their ability to manage their symptoms outside of their health care encounters. Sample items include “I can fit my medication schedule into my daily routine” (self-efficacy for managing treatment and medication) and “I can manage symptoms when I am at home” (self-efficacy for managing symptoms). Similar to all PROMIS measures, these measures are scored on a T-score metric with a mean of 50 and SD of 10. Higher scores indicate more of the construct being measured. These PROMIS measures have all demonstrated reliability, precision, and construct validity based on their correlation with legacy instruments [58].

##### Health-Related Quality of Life

To evaluate changes in health-related quality of life as an outcome associated with using the dashboard, a subset of health-related quality of life domains included in the symptom and goals assessment will be assessed again at the 3- and 6-month follow-ups (see Table 2 for a schedule of assessments). To assess changes in global health-related quality of life, patients will respond to the 7-item version of the FACT-G [48] in the baseline symptom assessment and in the 3- and 6-month follow-up surveys. The 7-item version of the FACT-G was derived from the 28-item version of the FACT-G [39] to offer a brief yet comprehensive assessment of multiple health-related quality of life domains relevant to patients with cancer. It has demonstrated adequate to good reliability, evidence of construct validity (convergent and known groups), and responsiveness to changes in health associated with an intervention [48,70]. To evaluate changes from baseline in core symptoms, the 3- and 6-month follow-up surveys will also contain computerized adaptive tests of the PROMIS anxiety [50], depression [50], fatigue [49], pain [51], and physical function [52] item banks.

##### Health Service Use

After study completion, we will retrospectively extract data on the use of potentially avoidable, high-cost, or low-value health care services from the periods 6 months before and 6 months after the first dashboard use. Specific indicators will include (1) unplanned all-cause hospital admissions, (2) potentially avoidable all-cause emergency department use, (3) excess (all-cause) days in acute care within 30 days of hospital discharge, and (4) 7-day readmissions. In addition, among patients with cancer, we will assess the following disease-specific indicators: (5) admissions and emergency department visits for patients receiving outpatient chemotherapy, (6) chemotherapy within the last 14 days of life, (7) use of a triage clinic, (8) completion of an advance directive, and (9) hospice use of >3 days. Among patients with chronic kidney disease, the following additional indicators will be collected: (10) use of emergency start dialysis; (11) chronic kidney disease–related emergency department or inpatient use; and (12) progression from chronic kidney disease stage 3 to stage 4, stage 4 to stage 5, or stage 3 to stage 5.

#### Implementation Outcomes

Patient- and clinician-reported survey measures will capture relevant implementation outcomes [42] of the demonstration trial supplemented by data from the electronic health record and qualitative data gathered by the study staff throughout the project. To assess the project’s *reach*, we will extract data from the electronic health record on patient enrollment rates in the demonstration trial and response rates to the symptom and goals assessments. To assess the *fidelity* of the dashboard’s use during the study period, patients will report whether the dashboard was discussed during their eligible clinical encounter in each dashboard visit evaluation survey, which is completed immediately after the appointment. Moreover, the study staff will conduct regular clinician observations to assess whether the dashboard is being used consistently and as intended. To assess *adoption*, clinicians will self-report in their 3- and 6-month surveys the frequency with which they use the dashboard with eligible patients. To assess *usability, acceptability,* and *adoption,* both patients and clinicians will rate the dashboard at the 3- and 6-month follow-ups on the System Usability Scale [66], the Stroke Prevention in Healthcare Delivery Environments study acceptability measure [67], and the Normalization Measure Development measure of adoption of new health care elements [68]. Finally, clinicians will rate the perceived *sustainability* of the clinical dashboard at the 6-month follow-up using the Clinical Sustainability Assessment Tool [69]. In addition to these implementation outcomes, we will closely document the implementation process, including strategies that were used to successfully carry out the project and implement the dashboard. These may include, but are not limited to, strategies for stakeholder engagement and co-design, patient recruitment and outreach, and training resources for using the dashboard to facilitate shared decision-making. Quantitative data will be analyzed using descriptive statistics, and qualitative data will be analyzed using the approaches described in objective 1.

#### Statistical Analyses

For all statistical tests, a nominal 2-sided *P* value of <.05 will be considered statistically significant. Unless otherwise noted, analyses will be conducted on the full analysis set and stratified by populations with chronic kidney disease versus cancer.

The primary analyses for objective 2 are within-group mean changes in shared decision-making (CollaboRATE), self-efficacy for managing chronic conditions (PROMIS), satisfaction with care (Functional Assessment of Chronic Illness Therapy-Treatment Satisfaction and Consumer Assessment of Health Plans Study Cancer Care Survey), and health-related quality of life and symptoms (PROMIS, Functional Assessment of Chronic Illness Therapy, and PRO-CTCAE). First, we will test whether within-group means have changed significantly from the baseline and symptom assessments (as appropriate) to the 3-month follow-up (primary) and 6-month follow-up (secondary) using paired samples *t* tests. We will supplement the paired *t* tests with multivariable regression models to adjust for key covariates (eg, demographic and clinical characteristics). Regarding health service use, we will first use paired-sample *t* tests to compare rates in the occurrence of each indicator described in the Health Service Use outcome section between the 6 months before and after the date of first dashboard exposure. On a secondary basis, we will model these outcomes as count variables using zero-inflated Poisson regression to adjust for covariates [71,72]. Finally, we will compare rates of health service use before and after the first dashboard exposure between patients exposed to the dashboard and the naïve comparison cohorts using 2-sample *t* tests and zero-inflated Poisson regression. To do so, we will use propensity score matching to create comparable cohorts of patients with and without exposure to the dashboard. We will match on age, sex, cancer type (where applicable), and disease severity or staging [73].

#### Power and Sample Size Calculations

Sample size considerations for objective 2 were informed by statistical power analyses for differences in within-group changes in mean responses to the CollaboRATE shared decision-making measure and PROMIS measures from baseline to the 3-month follow-up. Within-group mean changes in the CollaboRATE 5-point response scale version ranged from approximately 2.0 to 4.5, with SDs of approximately 3.5 [74]. For the CollaboRATE measure, we conducted a statistical power analysis making the following conservative assumptions: (1) a correlation between baseline and 3-month follow-up scores of *r*=0.30; (2) a common SD of 3.5 points; (3) a 2-sided *P* value of .05; and (4) mean changes of 1.0, 1.5, and 2.0 points. These analyses suggested that sample sizes of 137, 62, and 36 patients would be needed to detect mean changes of 1.0, 1.5, and 2.0 points, respectively, using a paired *t* test.

As noted in the Methods section, all PROMIS measures are scored as a T-score metric with a mean of 50 and SD of 10. Changes as low as 3 points can be clinically meaningful [75]. For power analysis regarding PROMIS measures, we made the following conservative assumptions: (1) a correlation between baseline and 3-month follow-up scores of *r*=0.50; (2) a common SD of 10.0 points; (3) a 2-sided *P* value of .05; and (4) mean changes of 3.0, 4.0, and 5.0 points. These analyses suggested that sample sizes of 90, 52, and 34 patients would be needed to detect mean changes of 3.0, 4.0, and 5.0 points, respectively, using a paired *t* test.

## Results

The co-design sessions (objective 1), which focused on collaboratively designing the dashboard’s content and format for initial clinical testing, concluded in May 2020. Focus groups supplementing the co-design sessions were completed in October 2019, February 2020, and October 2021. Data collection for the demonstration trial is anticipated to be completed by the end of July 2022. Study investigators currently meet monthly as an entire team to discuss progress on manuscript development for results dissemination. Each study objective will have at least one resulting publication: (1) co-design process and outcomes and (2) demonstration trial process and outcomes.

## Discussion

### Overview

In this study, we hypothesize that co-designing and implementing a patient-reported outcome clinical dashboard will be associated with improved processes underlying health care coproduction among adults with advanced cancer or chronic kidney disease, including shared decision-making, satisfaction with care, engagement in health care, self-efficacy in managing symptoms and treatments, health-related quality of life, and use of health services. Collaborating with patients, care partners, and clinicians to create scalable clinical dashboards can help improve coproduction outcomes [31,33]. Thus, this study draws upon a co-design framework to actively engage patients and care partners in the iterative design and evaluation of a patient-reported outcome dashboard to be used during clinical encounters with patients with advanced cancer and chronic kidney disease. However, few previous studies have attempted to improve the coproduction process by integrating patient-reported outcomes with other electronic health record–linked clinical results in a data visualization platform to facilitate shared decision-making [15,31,32]. In particular, our dashboard aims to improve recognition and shared decision-making for the management of patients’ symptoms and treatment needs, which can promote more effective use of the health care system and translate into improved patient outcomes [17,18,29,76].

### Strengths

The dashboard’s design and evaluation processes have several strengths worth noting. First, the dashboard will be co-designed by investigators and key stakeholders (patients, care partners, and clinic staff). Collaborating with stakeholders promotes person-centered care delivery and increases the likelihood that a new health care element can be successfully implemented and optimally impactful [33]. Unfortunately, stakeholder engagement is often skipped when developing interventions owing to time and money constraints [77]. Second, we will carefully document each step of the dashboard design and implementation process, including any unanticipated modifications needed for improving its acceptability, feasibility, and adoption. This will ensure that the dashboard can be easily disseminated to other investigators and adopted by other health care systems and that the study can be replicated. Third, the dashboard is designed to be dynamic and individualized. It will be fully integrated within the electronic health record, providing a more complete picture of a patient’s health status and facilitating informed decision-making and treatment planning by displaying patient-reported outcomes along with other clinical data. New data populate the dashboard in real time so that health care can efficiently adapt to patients’ changing symptom and care needs. Fourth, patients can view the dashboard at any time through their electronic patient portal, which will display symptom scores over time in user-friendly graphs. This will allow patients who do not complete regular health care visits to still experience benefits through enhanced self-reflection and self-monitoring of personalized symptom feedback even if explicit health care decision-making does not occur. In summary, our patient-centered dashboard and design process provide an efficient, feasible, and scalable model for integrating patient-reported outcome data collection into person-centered, value-based care delivery.

### Limitations

Despite these strengths, this project has limitations that warrant discussion. First, the study will focus only on patients in the advanced stages of 2 chronic conditions and on those who regularly access their electronic patient portals. Although advanced cancer and chronic kidney disease are two of the most common and burdensome illnesses to individuals and the health care system [3,21,23], we are actively expanding the dashboard into other areas of medicine such as rheumatology. Second, the electronic health record software in which the dashboard will be developed has limitations in how data are visualized, which limits the opportunities for user-centered design. Nonetheless, integration into the electronic health record will allow us to improve other aspects of the user experience, including reducing the clinical burden that might be associated with using an external system, providing a shared dashboard environment that is accessible to both patients and clinicians, and allowing patient-reported outcome results to be integrated with other routinely collected health information. Third, the dashboard will be evaluated using a single-arm design, precluding the ability to draw causal conclusions about any observed changes in patient outcomes during the demonstration project. Fourth, the dashboard and study process will only be available in English and may underrepresent people with low health literacy and less digital acumen or computer access. Future efforts will need to adapt the dashboard to other languages, chronic illness groups, and health ITs represented in the local health care system. There may also be an opportunity in the future to conduct a formal randomized controlled trial of the dashboard’s efficacy.

This study demonstrates how to combine stakeholder engagement, a health care coproduction framework, and health IT to develop a clinically feasible, acceptable, and scalable model of patient-centered care delivery. This study’s premise integrates emerging research indicating that better patient outcomes and appropriate health care use can be enhanced through patient-clinician collaboration in the context of well-designed workflows and systems. Thus, this study focuses on dealing with the manifold challenges of implementing significant changes in workflow and clinical approach in the real world of ambulatory care of patients with a serious chronic illness. We aim to build on implementation research to understand the needed adoption, acceptability, and feasibility of new tools to optimize a successful and sustainable transformation of the clinical encounter over time. Our work serves as an example for transforming care delivery in a way that embraces value-based care and has the potential to improve the lives of patients with chronic conditions.

## Abbreviations

FACT-G: Functional Assessment of Cancer Therapy-General
PRO-CTCAE: Patient-Reported Outcomes version of the Common Terminology Criteria for Adverse Events
PROMIS: Patient-Reported Outcomes Measurement Information System
REDCap: Research Electronic Data Capture

## Data Availability

Although we have collected data for this study, this manuscript does not report any results. Our intention is to share data associated with the published results as they emerge.
